# Relevance of the anterior cingulate cortex volume and personality in motivated physical activity behaviors

**DOI:** 10.1038/s42003-023-05423-8

**Published:** 2023-10-31

**Authors:** Anna Miró-Padilla, Jesús Adrián-Ventura, Anastasia Cherednichenko, Irene Monzonís-Carda, Maria Reyes Beltran-Valls, Diego MolinerUrdiales, César Ávila

**Affiliations:** 1 Neuropsychology and Functional Neuroimaging Group, Department of Basic Psychology, Clinical Psychology and Psychobiology, Universitat Jaume I, Castelló de la Plana, Spain; 2 Department of Psychology and Sociology, University of Zaragoza, 44003 Teruel, Spain; 3 LIFE Research Group, Department of Education, Universitat Jaume I, 12071 Castellon, Spain

**Keywords:** Reward, Personality

## Abstract

Some recent theories about the origins and maintenance of regular physical activity focus on the rewards of the properties of practicing this activity. Animal and human studies have demonstrated that mesolimbic dopamine plays a crucial role in the involvement in voluntary physical activity. Here, we test this possible role in a sample of 66 right-handed healthy young adults by studying the influence of personality and the volume of reward-related brain areas on individual differences in voluntary physical activity, objectively measured by accelerometer and subjectively self-reported by questionnaire. Our results show that a smaller volume of the right anterior cingulate cortex and lower scores on reward sensitivity contributed to explaining low levels of daily physical activity. Moreover, the volume of the right anterior cingulate cortex correlates positively with self-reported total physical activity. Results are discussed by highlighting the need to use objective measures of daily physical activity, as well as the important role of the anterior cingulate cortex and personality in promoting effortful and invigorating actions to obtain rewards.

## Abstract (web summary)

In a cohort of right-handed young adults, the volume of the right anterior cingulate cortex positively correlates with self-reported physical activity.

## Introduction

High levels of daily physical activity (PA) has multiple benefits for physical health and acts as a protector against psychological (e.g., depression and anxiety) and neurodegenerative (e.g., Parkinson’s disease) illnesses^1,2^. In addition, research has demonstrated that PA has a positive impact on cognitive function^3^, given that athletes present faster and more efficient visuo-spatial and memory processing^4^ and show greater cognitive flexibility and executive control^5,6^. Nevertheless, despite these benefits, 27.5% of adults do not achieve recommendations of 150–300 min of moderate PA per week, as stated by the World Health Organization (WHO^6^). In light of this, what factors would predispose adults to maintaining an active or sedentary lifestyle? Unfortunately, these factors are still unknown^7^. However, it is well known that PA practice is an accepted reinforcer for some individuals^8^, and it is possible that personality traits have a strong influence on these individual differences^7^. Consequently, in order to establish effective PA promotion programs, it would be of paramount importance to understand the mechanisms—subject to individual differences—associated with behavioral regulation in relation to daily PA. This information would be extremely relevant in choosing promotion techniques focused on establishing goals and using selfregulatory strategies^9^.

The most widely employed method to assess individual differences in PA and sedentary behaviors in previous scientific psychological studies is self-reported questionnaires. However, these measures have some limitations in terms of concurrent and criterion validity and reliability^10,11^. Reviews show that test-retest reliability in short periods is moderate to high. Regarding concurrent validity, the various self-reported measures designed to tap individual differences in PA have moderate to high correlations with each other, although lower for moderate PA than for vigorous PA^12^. Criterion validity studies that compare self-reported questionnaires with objective measures obtained from accelerometers have only yielded low to moderate correlations^13^.

Several investigations have shown that PA practice can induce structural changes in the brain, but most of these studies have focused on late adulthood, reporting higher levels of gray matter (GM) and white matter (WM) volume in different cerebral areas^14–18^. For example, neuroimaging studies in healthy elderly people have found a consistent positive association between PA and greater hippocampus and prefrontal cortex volume^15,19,20^. The scarce literature relating PA and cerebral volume in non-athlete young adults seems to indicate increased volume and functional activity of the hippocampus in physically active people^21–23^. Research evaluating PA and cerebral volume has mainly focused on the hippocampus^17,18^, ignoring other regions of interest that could be influenced by daily PA levels.

One of the complementary views for understanding individual differences in the amount of PA is the motivational approach^24^. This view stems from the intrinsic motivational and reinforcing properties of this behavior, given that engagement in effortful PA may be considered a voluntary choice, to the detriment of other activities such as a sedentary lifestyle. In this choice, the reward system and the mesolimbic dopaminergic pathways play a crucial role. This reward circuit is responsible for promoting effortful behaviors to attain rewards, leading the person to perform actions in order to approach potential reinforcers^8^. Animal studies clearly demonstrate that dopamine depletion in reward brain areas diminishes the probability of engaging in effortful activities such as wheel-running by increasing the preference for less-effortful activities such as sucrose intake^25,26^. Thus, the initiation and maintenance of different levels of PA may depend on the structure and functioning of the reward system. In fact, some studies have shown that nucleotide polymorphisms related to the reward system predispose humans to practicing different types of effortful activities^27,28^.

Three brain areas are mainly involved in evaluating secondary rewards such as PA: orbitofrontal cortex (OFC), anterior cingulate cortex (ACC), and nucleus accumbens (NAcc)^29^. These brain areas mediate decision-making in rewarded and goal-directed behaviors by evaluating the reward value and the choice of response alternatives and predicting the risk of each behavior. The NAcc plays a pivotal role in detecting possible reward stimuli. Once detected, the OFC evaluates the reward value and magnitude of the stimulus, promoting the reward-directed behavior as a function of the probability of obtaining it. Finally, the ACC detects and monitors possible conflicting situations when responding to achieve a reward. Recent views of the ACC have focused on its specific role in effortful control of invigorating actions that lead to rewards^30,31^.

Interestingly, individual differences in the activity of the human reward system can be studied based on personality traits. The reinforcement sensitivity theory (RST^32^) offers a neuropsychological research framework for studying variations in reward-related motivated behaviors^33,34^. The RST establishes the Behavioral Activation System (BAS) as a neurobehavioral system rooted in the reward system. The sensitivity and reactivity of this system can be tracked from stable personality traits and specific measures such as sensitivity to reward (SR^35^) or the BAS scale^35^. As such, individual differences in the activity of the reward system constrain a behavioral repertoire characterized by more vigorous and frequent actions aimed at obtaining rewards, with practicing PA being a good example of this behavior. Structural Magnetic Resonance Imaging (MRI) studies have negatively linked BAS-related traits to the striatal GM volume^36–38^ and the volume and cortical thickness of medial prefrontal regions such as the ACC^38,39^. Furthermore, these BAS-related traits have also been associated with a different connectivity pattern at rest within the reward system, modulating the connectivity between key areas such as the striatum, ACC, and OFC^40,41^.

As a rewarding activity, we would expect SR to be positively related to engagement in PA. However, the relationship between SR and PA is more complex. A recent study demonstrated that SR was associated with exercise dependence symptoms, that is, a predisposition to exercise addiction^42^. However, SR also predisposes to different behaviors such as drug addictions^43^, behavioral addictions^44^, or obesity^45^, which act against the probability of being involved in regular PA and could predispose to a sedentary lifestyle. The different studies that have directly investigated the relationship between SR and health-related behaviors have shown inconclusive results because they found no relationship in adults^46^ and children^47^ but a positive relationship in adults^48^.

Some studies have investigated the effect of physical condition on the human reward system. One of the most important studies revealed a positive association between the volume of ACC and OFC and measures of cardiorespiratory fitness in a large sample of healthy adults between 21 and 84 years old (mean 52.3)^49^. Two different studies have reported that exercise training increased the volume of the ACC in sedentary older adults^20,50^. Moreover, some studies have reported a positive correlation between the basal ganglia volume and fitness measures in preadolescents^51^ and young adults^52^_._ In the same vein, measures of fitness improvement after training have been correlated with increased perfusion in the ventral striatum^53^, whereas studies in patients with Parkinson’s disease reported an increased dopamine release in the ventral striatum due to exercise^54,55^. Thus, all the studies have reported positive correlations between volume or perfusion measures of reward-related structures and physical condition in samples that include sedentary and regular exercisers. However, a recent study showed a negative correlation between exercise addiction measures and the volume of the OFC, suggesting that extensive and vigorous PA may change the global pattern of results^56^.

Therefore, the main aim of this study was to investigate the relationship between the GM volume in key cerebral areas related to the reward system and intensity levels of daily PA in a sample of healthy university students using both objective (i.e., accelerometers) and subjective (i.e., questionnaires) measurements. We chose the OFC, NAcc, and ACC due to their aforementioned implication in effort-based decision-making and reward-related motivated behaviors^24,57,58^. In addition, personality traits were studied to determine whether the GM volume and personality traits jointly predicted PA. To this end, a voxel-based morphometry (VBM) analysis approach was used to investigate the possible differences in GM volume in a sample of 66 healthy young adults. We have focused on this age period because we can better capture the individual differences in PA for two different reasons. First, this is the first period in which physical activity is not mandatory for educational reasons. Second, developmental studies have shown that the structural and functional connectivity in the reward system is qualitatively different when compared adolescents to adults^59,60^.

Predictably, we expected a positive association between GM volume in reward-related structures and time of light/moderate PA and, on the contrary, a negative association between GM volume in these structures and sedentary time. We also explore the mediating role of personality measures of reward traits in this relationship. This analysis provides information about the brain effects of PA associated with personality traits, given that, to the best of our knowledge, no studies to date have compared objectively measured PA and GM volume in young adults.

## Results

Descriptive statistics for all the variables included in the study are shown in Table 1.

**Table 1:** Descriptive statistics included in the study (*N* = 66).

	M	SD	Min.	Max.
Age (years)	22.70	2.898	18	29
Total GM (ml)	734.16	61.05	621.41	881.84
Height (cm)	171.04	9.96	151	190.2
Weight (kg)	67.08	14.78	38.25	130.2
BMI (kg/m^2^)	22.8	4.00	16.78	38.29
Abdominal Circumference (cm)	77.24	10.04	57	116.5
Objectively measured PA (min/day)				
Sedentary time	775.34	74.09	610.20	994.33
Light	154.3	49.78	57.87	315.14
Moderate	54.26	20.58	11.92	95.83
Vigorous	2.30	2.15	0.14	8.76
Moderate and vigorous	56.56	21.37	12.06	100.65
Self-reported measured PA (min/day)				
Sedentary	415.69	230.01	60	1200
Moderate and vigorous	621.52	515.22	51	2229
Moderate	689.12	651.80	0	2880
Vigorous	229.81	263.08	0	1051
Personality traits				
Sensitivity to Punishment	11.15	5.15	1	24
Sensitivity to Reward	10.65	4.23	3	22
Behavioral Inhibition System	21.52	3.33	12	28
Behavioral Activation System	42.0	4.9	28	52
ROIs’ GM volume (in ml)				
Right Anterior Cingulate Cortex	3.72	0.49	2.81	5.02
Left Anterior Cingulate Cortex	4.64	0.58	3.41	6.09
Right Nucleus Accumbens	0.45	0.05	0.36	0.62
Left Nucleus Accumbens	0.5	0.06	0.37	0.68
Right Orbital Frontal Cortex	11.11	1.19	8.86	14.92
Left Orbital Frontal Cortex	11.13	1.11	8.7	14.63

*GM* gray matter, *BMI* Body mass index, *ROI* region of interest, *PA* physical activity.

We calculated Spearman’s correlations between the PA measures to report the correspondence between them, and these correlations appear in Table 2. We found moderate significant correlations between the self-reported and objective measures that were stronger for vigorous activity than for light-moderate activity.

**Table 2:** Spearman’s correlations between objective and subjective PA measures.

		Objectively measured PA			Self-reported PA		
		Sedentary	Light	MVPA	Sedentary	MVPA	Vigorous
Objectively measured PA	Sedentary		−0.37**	−0.34**	0.17	−0.12	0.04
	Light			0.70**	−0.30*	0.23	0.17
	MVPA				−0.26*	0.29*	0.35**
Self-reported PA	Sedentary					−0.40**	−0.26*
	MVPA						0.69**
	Height	0.24	−0.22	−0.12	−0.09	0.11	0.04
	Weight	0.15	−0.27*	−0.09	04	0.18	0.01
	BMI	0.03	−0.20	−0.04	0.10	0.14	0.05
	Abdominal circumference	0.10	−0.33**	−0.11	−0.02	0.08	0.08

*BMI* body mass index, *PA* physical activity, *MVPA* moderate and vigorous PA.

*p < 0.05, **p < 0.01.

Table 3 shows the correlations between the personality and PA measures. Pearson’s correlations were run between the accelerometer-derived PA measures and the personality traits, whereas Spearman’s correlations were used for GPAQ-derived PA measures. Results showed that the BIS-related measures were related to less self-reported vigorous PA and less sedentary behavior measured with the accelerometer.

**Table 3:** Partial correlations between objective and subjective PA and personality measures (controlling for age and total GM volume).

		Objectively measured PA			Self-reported PA		
		Sedentary	Light	MVPA	Sedentary	MVPA	Vigorous
Personality measures	SP	−0.06	0.10	−0.037	0.20	−0.27*	−0.41**
	SR	−0.06	−0.02	−0.06	−0.10	0.06	0.10
	BIS	−0.27*	0.16	0.00	0.11	−0.22	−0.38**
	BAS	−0.21	0.00	0.06	−0.32*	−0.00	−0.07

*MVPA* moderate and vigorous PA, *SP* Sensitivity to Punishment, *SR* Sensitivity to Reward, *BIS* Behavioral Inhibition System, *BAS* Behavioral Activation System.

*p < 0.05, **p < 0.01.

**Fig. 1:**
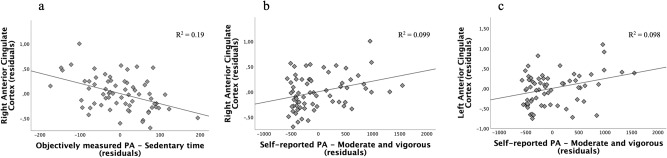
Representations of significant correlations with PA measures and the volume of the Anterior Cingulate Cortex. Scatterplots of residuals illustrating the relationship between: **a** sedentary time objectively measured and the volume of the Right Anterior Cingulate Cortex; **b** moderate and vigorous PA self-reported measure and the volume of the Right Anterior Cingulate Cortex; and **c** moderate and vigorous PA self-reported measure and the volume of the Left Anterior Cingulate Cortex.

To test the main hypothesis of this study, which proposed a relationship between the brain volume in reward-related structures and a stronger predisposition to global PA, we ran partial correlations between the volume of the six reward region-of interest (ROIs) and the measures of sedentary behavior and global PA, including age and total GM as nuisance covariates Results appear in Table 4 and in Fig. 1. A negative association was found between objective sedentary time and the GM volume of the right ACC (*r* (62) = −0.44, *p* < 0.001). Moreover, a positive association was found between self-reported MVPA and the volume of the right ACC (*r* (62) = 0.32, *p* < 0.05) and left ACC (*r* (62) = 0.31, *p* < 0.05). Correlations for the left and right OFC and NAcc were not significant in any case.

**Table 4:** Partial correlations between objective and subjective PA and the volume of the reward ROIs (controlling for age and total GM volume).

		Objectively measured PA		Self-reported PA	
		Sedentary	Light	Sedentary	MVPA
Reward ROIs	Right Anterior Cingulate Cortex	−0.44**	0.06	0.01	0.32*
	Left Anterior Cingulate Cortex	−0.13	0.07	0.02	0.31*
	Right Nucleus Accumbens	0.06	−0.01	−0.16	0.04
	Left Nucleus Accumbens	0.04	0.01	−0.20	0.12
	Right Orbital Frontal Cortex	0.07	0.05	−0.03	0.01
	Left Orbital Frontal Cortex	0.00	−0.07	0.05	−0.00

^*^p < 0.05; **p < 0.01.

Finally, multiple regression analyses were conducted to examine possible additive effects of the GM volumes and personality on the PA measures. We obtained a significant model when sedentary time was taken as the dependent variable. The right ACC volume, GM, age, and BAS were included in the model (adjusted *R*² = 0.23; *F*_4,61_ = 5.90; *p* < 0.001). The beta values for the right ACC volume, GM volume, age, and BAS were −0.62, 0.62, −0.14, 366, and −0.23, respectively.

## Discussion

In the present study, VBM analysis was used to investigate the relationship between PA and personality in key cerebral areas related to the reward system (OFC, ACC, and NAcc) in a healthy young population. Self-reported and objective (accelerometer) measures of daily PA levels were utilized. Two significant correlations were found, a negative one between the accelerometer measure of sedentary time and right ACC GM volume, and a positive one between self-reported MVPA and right ACC GM volume. Both results indicate that the longer the sedentary time, the lower the volume of the right ACC. Moreover, the regression analysis showed that the BAS activity and the right ACC GM volume are negatively related to individual differences in sedentary time. Thus, both personality and anatomical factors contribute separately to explaining the proneness to inactivity.

Accelerometer measures of PA showed low to moderate correlations with the GPAQ measures, as a recent meta-analysis found^13^. The strongest correlation was obtained with vigorous activity, but it was not significant for moderate activity or sedentary time. Crucially, in our neuroimaging results, the objectively measured sedentary time obtained with the accelerometer did not correlate with self-reported sedentary time, but it did correlate negatively with activity time. The factors explaining the discrepancy between subjective and objective measures may be related to the differences in the registration period (one week for accelerometers, but less specific for GPAQ) and the subjective interpretation of the different items typically observed in self-reported questionnaires. In this regard, previous data have shown that the body mass index may bias the response to these questionnaires^61^. Our data show negative correlations between light PA measured with the accelerometer and BMI and weight measures. However, these correlations were not observed for the GPAQ-derived measures. It is possible that some individuals were less likely to report PA due to social desirability.

The present investigation shows that the ACC volume is related to PA and sedentary time in healthy young adults, a result that had mainly been reported in the elderly and middle-aged population to date^20,49,50^. All these results point in the same direction: the more PA, the greater the ACC volume observed. Interestingly, a recent structural MRI study in young adults associated the ACC with PA^62^. The authors reported that greater ACC thickness was associated with a larger daily volume of MVPA and higher levels of cardiorespiratory fitness. In this research, we have gone a step further by showing that the ACC volume was associated with sedentary time and MVPA measured with both objective and subjective measures, respectively.

The ACC is considered a neural structure involved in a variety of cognitive functions related to motivational control, such as error detection, conflict monitoring, response selection, attention and task preparation, reward prediction, effort prediction, or perception of pain^30^. Recent views of the role of the ACC focus on the concept of effort, that is, on the capacity to adequately select behaviors and activities that require effort by combining the information about internal motivation and required task effort^63^. Consistent with this view, a recent study using a large sample related a smaller ACC volume to anhedonia^64^. In our study, the ACC volume was negatively related to objectively-measured sedentary behavior and positively to self-reported MVPA, that is, the probability of being involved in effortful behavior to increase health. In this line, previous studies have negatively related the ACC to obesity^65,66^ and an increased proneness to behavioral and drug addictions^67–72^. Thus, the volume of the ACC seems to be positively related to a higher probability of selecting and enacting behaviors that lead to greater long-term gains over short-term gains^73,74^. Likewise, a recent study with a large sample showed that the volume of the ACC correlated with a stronger internal locus of control, a personality trait related to subjective and physical well-being, self-efficacy, emotional stability, and health^75^. The overall data seem to tentatively suggest that an increased ACC volume would be a predisposing factor to learning habits that improve health and self-care.

The only significant direct correlation between personality and PA was found between both SP measures and fewer minutes dedicated to self-reported VPA, which is consistent with previous data showing that this personality trait was negatively associated with exercise dependence symptoms^42^. Our results showed that measures of reward sensitivity were not directly related to PA measures. This is consistent with previous studies in adults and children^46,47^ and may indicate that BAS activity also predisposes people to other addictive behaviors more compatible with sedentarism^47,76,77^. However, our regression analysis revealed that the BAS subscale significantly contributes, along with the right ACC volume, to explaining objectively measured sedentary time. This is crucial for understanding the concept of reward sensitivity (BAS activity) because it may imply that this trait contributes to the emission of rewarding motivated behaviors such as PA in individuals with a larger ACC volume and a greater predisposition to subjective and physical well-being.

In conclusion, the current study showed that sedentary time is negatively associated with right ACC volume in healthy young adults, and that personality traits have an influence on this relationship. These findings will contribute to adding evidence about the effects of PA on the brain in a healthy young population. In addition, they may have relevant implications from public health perspectives when designing lifestyle programs adapted to the personality traits of each person, such as mental contrasting^9^. Thus, our results would serve to hypothesize that the efficiency of such intervention programs may vary as a function of ACC volume and reward sensitivity.

## Methods

### Participants

A total of 66 right-handed, healthy young adults (33 females) with ages ranging between 18 and 29 years (mean age = 22.7; SD = 2.9) participated in the study. They were recruited from the student community of Universitat Jaume I, and none of them had a previous psychiatric or neurologic diagnosis. As an inclusion criterion, all participants had a scaled score >8 on the matrix reasoning test from the WAIS-III. Height, weight, Body Mass Index (BMI) and abdominal circumference were calculated for all participants for descriptive purposes. Informed consent was obtained from each subject before participation, and they received monetary compensation for their time and effort. The Ethical Committee of Universitat Jaume I approved the research project (CD/11/2021).

### Measures

A GENEActiv accelerometer (Activinsights Ltd, Kimbolton, UK) was used to measure PA and sedentary time. This waterproof device contains a triaxial microelectromechanical accelerometer that records both motion-related and gravitational acceleration and has a linear and equal sensitivity along the three axes. All participants wore the accelerometer on their non-dominant (left) wrist. Accelerometer-derived data from all participants comprised at least four days, including weekend and weekdays, with 24 h valid data. Devices were programmed with a sampling frequency of 100 Hz, and data were stored in gravity (g) units (1 g = 9.81 m/s^2^). The raw acceleration output was converted to 1-s epochs using the GENEActiv Post-Processing PC Software, version 2.2. By combining all the registered days for each participant and according to ref. ^78^, PA was expressed as the average (min/day) of sedentary, light, moderate, and vigorous PA. Time spent in moderate and vigorous PA (MVPA) was determined by adding minutes per day of moderate and vigorous PA. The GENEActiv cut off point for vigorous PA was established for values ≥ 1810 g, moderate PA for values between 645 and 1810 g, light PA for values between 217 and 644 g, and sedentary time was established for values ≤ 217 g.

In addition, all participants completed the self-reported Global Physical Activity Questionnaire (GPAQ) published by the World Health Organization (WHO; https://www.who.int/ncds/surveillance/steps/GPAQ_ES.pdf) as part of the WHO STEPwise Approach to Noncommunicable Disease Risk Factor Surveillance. Based on the responses, the total metabolic equivalent task (MET) minutes per day for sedentarism, moderate and vigorous PA were calculated for each participant. In the same way, as with the objectively measured PA, time spent in MVPA was calculated by adding minutes per day of moderate and vigorous PA.

The Sensitivity to Reward Scale (SPSRQ)^34^ and the BAS scale^35^ were used to assess individual differences in BAS activity. These self-reported questionnaires measure individual differences in sensitivity to various rewards, including reinforcers such as money, sexual partners, social recognition, power, or loss of sensations, which describe heterogeneous situations in promoting responses to obtain rewards. Additionally, we also included the Sensitivity to Punishment (SP) and the Behavioral Inhibition scales (BIS) from the same questionnaires in order to explore possible effects derived from behavioral inhibition traits on the PA practice.

### MRI acquisition and image preprocessing

Anatomical MRI data were acquired using a 3 T General Electric Signa Architect scanner (Waukesha, WI, USA). A high-resolution T1-weighted BRAVO sequence was acquired, covering the whole brain (TE  =  3.28 ms, TR  =  8.52 ms, FOV = 240 mm, phase FOV = 100%, flip angle = 12°, inversion time = 450 ms, matrix = 256 × 256, voxel size = 0.5 × 0.5 mm, space between slices = 0.5 mm, slice thickness = 1 mm, number of images = 384, sequence length = 4:17 min). A 24-channel coil was used, whereas the scan plane angulation was strictly sagittal. Participants were placed in a supine position inside the MRI scanner, and their heads were immobilized with pads to reduce involuntary motion. Additionally, landscape images were presented during the sequence run through MRI compatible goggles (VisuaStim, Resonance Technology Inc., Northridge, CA, USA).

VBM was conducted by means of the Computational Anatomy Toolbox (CAT12; v12.8, r1885; www.neuro.uni-jena.de/cat/) for the Statistical Parametric Mapping software (SPM12; v7771; www.fil.ion.ucl.ac.uk/spm/software/spm12/) under Matlab R2018b (v9.5). All the T1-weighted images were first reoriented to the anterior–posterior commissure line and subsequently preprocessed by means of the CAT12 segment module. Following the standard preprocessing pipeline, we implemented all the steps recommended in the manual: 1) segmentation of the original images into GM, WM, and cerebrospinal fluid by using the tissue probability maps included in SPM; 2) affine registration and regularization based on the space template provided by the International Consortium for Brain Mapping (ICBM); 3) normalization (warping) of the GM segments to the Montreal Neurological Institute (MNI) template via DARTEL; and 4) modulation by the affine + non-linear components (SPM default) derived from the spatial MNI normalization. Then, once the preprocessing was completed, a quality check of the resulting data was conducted by means of the CAT12 check sample homogeneity of 3D data module. After including all the quality measures obtained during the segmentation, as well as total intracranial volume (TIV; obtained via the get TIV module) and age as nuisance variables, the results showed no potential outliers in brain volume. Finally, prior to the statistical analyses, all the resulting modulated, normalized GM maps were spatially smoothed with an 8-mm full-width at half-maximum Gaussian kernel.

### Statistical and reproducibility

**Fig. 2:**
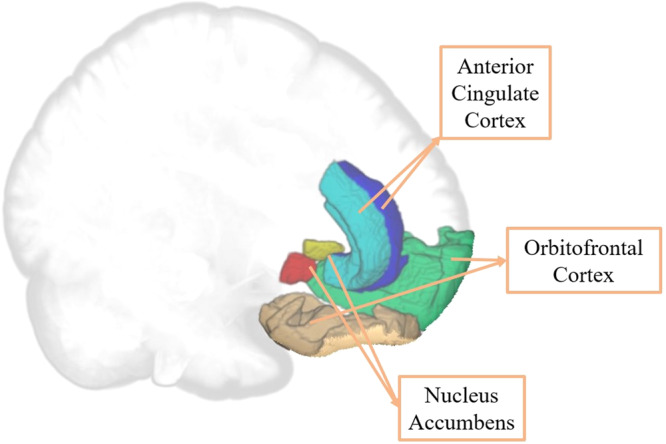
Selected key ROIs in the reward system (extracted from the Neuromorphometrics atlas).

Given that the main aim of the present study was to examine the differences in GM volume in the reward system as a function of PA, ROI analyses were conducted for the whole sample (*N* = 66). The CAT12 standard pipeline (ROI Tools module) was followed: 1) estimating the mean GM volume (in ml) of each region included in the Neuromorphometrics atlas for each participant in native space; and 2) selecting six bilateral key ROIs in the reward system: left and right OFC, nAcc, and ACC (see Fig. 2). The Neuromorphometrics atlas divides the OFC into four parts; in order to explore the entire OFC, the GM volumes of all the parts were added.

Afterwards, volumetric and PA data were computed with the IBM SPSS Statistics for Windows, Version 27.0 (Armonk, NY: IBM Corp), for statistical proposes. Spearman or Pearson correlations were performed, controlling for age and total GM volume: 1) between objective and subjective PA measures; 2) between personality and PA measures; and 3) between the volume of the ROIs and PA measures. In addition, hierarchical multiple regressions were run to determine whether the ROIs’ GM volume and personality traits predicted PA, using the PA measures (objective and self-reported) as dependent variables. In the first step, age and total GM volume were introduced in the model using the enter method. In the second step, personality measures (SP, SR, BIS, and BAS) and the ROIs’ GM volume were introduced in the model using the stepwise method. The stepwise method was adopted in order to extract only significant (*p* < 0.05) predictors. Our regression analysis was restricted to the PA measures and ROIs that correlated significantly.

### Reporting summary

Further information on research design is available in the Nature Portfolio Reporting Summary linked to this article.

### Supplementary information

Description of Additional Supplementary Files

Supplementary Data

Reporting Summary

## Data Availability

Processed data to reproduce our main findings is available on the web page https://drive.google.com/file/d/19Cjn5pQdajx5JkA6ennEHjVRYH2Se_Q-/view. The MRI images available from the corresponding author on reasonable request. The source data for Tables 1–4 and Fig. 1 can be found in Supplementary Data.

## Code availability

All the codes supporting this study are available on www.neuro.uni-jena.de/cat/ and www.fil.ion.ucl.ac.uk/spm/software/spm12/. The custom codes that support the findings of this study are available from the corresponding author or first author upon reasonable request.
