# Supplementary material for: Relevance of the anterior cingulate cortex volume and personality in motivated physical activity behaviors
Source: Commun Biol. 2023 Oct 31;6:1106. doi: 10.1038/s42003-023-05423-8 (PMC10618534; doi:10.1038/s42003-023-05423-8)
Supplement: Supplementary file 1 — Description of Additional Supplementary Files [file 42003_2023_5423_MOESM1_ESM.pdf]

Description of Additional Supplementary Files

File name: Supplementary Data

Description: Source data for Tables 1-4 and Figure 1 included in the paper.
